# Complex Fluids in a Multifractal Space: Scale Covariance and the Emergence of the Fractal Force

**DOI:** 10.3390/e28020189

**Published:** 2026-02-09

**Authors:** Dragos-Ioan Rusu, Vlad Ghizdovat, Lacramioara Ochiuz, Oana Rusu, Iuliana Oprea, Lucian Dobreci, Maricel Agop, Decebal Vasincu

**Affiliations:** 1 Department of Environmental Engineering, Mechanical Engineering and Agritourism, Faculty of Engineering, “Vasile Alecsandri” University of Bacau, 600115 Bacau, Romania; drusu@ub.ro (D.-I.R.);; 2 Department of Biophysics and Medical Physics-Nuclear Medicine, Grigore T. Popa University of Medicine and Pharmacy Iasi, 700115 Iasi, Romania; 3 Faculty of Material Science and Engineering, “Gheorghe Asachi” Technical University, 700050 Iasi, Romania; 4 Department of Mathematics, Colorado State University, Fort Collins, CO 80523, USA; juliana@math.colostate.edu; 5 Department of Physical and Occupational Therapy, “Vasile Alecsandri” University of Bacau, 600115 Bacau, Romania; 6 Academy of Romanian Scientists, 3 Ilfov, 050044 Bucharest, Romania

**Keywords:** multifractal dynamics, complex systems, self-organization, scale covariance

## Abstract

Complex systems—ranging from biological organisms to turbulent fluids—exhibit multiscale heterogeneity and intermittency that traditional, differentiable calculus fails to adequately capture. Therefore, we propose a mathematical framework for analyzing complex system dynamics by assimilating the trajectories of structural units to continuous but non-differentiable multifractal curves. Utilizing the scale covariance principle, the authors recast the conservation of momentum as a geodesic equation within a multifractal space. This approach naturally separates the complex velocity field into differentiable and non-differentiable scale resolutions, where the balance of multifractal acceleration, convection, and dissipation is parametrized by a singularity spectrum *f*(*α*). We also discuss broad interdisciplinary implications, because, in our opinion, non-differentiability can enhance predictive capabilities in various fields such as oncology, pharmacology, and geophysics.

## 1. Introduction

Complex systems constitute a central explanatory framework for phenomena in which coherent macroscopic organization arises from the interactions of many components. Across disciplines, the most consequential objects of study, such as cells, organisms, brains, ecosystems, turbulent flows, and infrastructural or social networks, cannot be adequately understood by decomposing them into isolated parts and recombining linear effects. Instead, collective behavior is shaped by feedback, heterogeneity, and multiscale coupling between processes operating at different spatial and temporal scales, so that system-level regularities must be explained in terms of interaction rules and their dynamical consequences rather than in terms of component properties alone [1].

A single universally accepted definition of a “complex system” is elusive, in part because complexity is not a property of size alone but of interaction structure and dynamical regime. A widely used working definition treats a complex system as an ensemble of many heterogeneous units (agents, degrees of freedom, nodes) whose interactions are sufficiently rich and nonlinear that system-level patterns (spatiotemporal regularities, functions, or information-processing capabilities) emerge from the bottom up and are not straightforwardly deducible from isolated component behavior. In the specific case of complex adaptive systems, the components can modify their interaction rules through learning, selection, or adaptation, creating an additional feedback loop between micro-level behavior and macro-level organization [2,3].

Several properties recur across complex systems and organize both empirical investigation and theory. Emergence and self-organization describe the appearance of collective order without centralized design. In nonequilibrium settings, sustained fluxes of energy or matter can move systems into regimes where instabilities amplify fluctuations and generate organized macrostates (“dissipative structures”) [4].

Complex systems are also typically multiscale and only partially decomposable. Simon’s notion of near-decomposability highlights that subsystems may have comparatively fast internal dynamics relative to slower interactions between subsystems, creating an apparent hierarchy that supports coarse-graining while retaining essential coupling [5]. Anderson’s argument that “more is different” similarly stresses that new organizing principles and effective laws become relevant at higher levels of aggregation, so that reductionism alone is insufficient for explaining collective phenomena [6]. These perspectives motivate systematic links between levels of description: identifying variables appropriate for a given scale, determining when lower-level details can be averaged out, and characterizing when cross-scale feedback renders such averaging invalid.

Nonlinearity further implies that sensitivity and robustness can coexist. Instabilities, chaotic dynamics, and bifurcations can produce disproportionate responses to perturbations, yet biological and engineered complex systems often remain functional under noise, damage, and parameter variation through redundancy, degeneracy, and distributed control. This coexistence motivates robustness as an emergent system property rather than an attribute of any single component [7]. In complex adaptive systems, robustness and adaptability are linked: components can modify behavior in response to environmental changes, and such adaptation may alter global regimes, sometimes stabilizing function and sometimes creating new vulnerabilities [2,3].

Interaction topology is a further determinant of complex-system behavior, and network science provides a compact representation of this topology. Components correspond to nodes and interactions to edges, enabling comparable analysis across biochemical reaction graphs, neuronal circuits, ecological food webs, and technological infrastructures [8,9]. Real networks are rarely well approximated by regular lattices or homogeneous random graphs; instead, they tend to be sparse yet structurally heterogeneous, with clustering, modular community structure, and broad degree distributions [9,10]. These motifs are not merely descriptive: they constrain controllability, robustness, and the propagation of perturbations, information, and pathogens, and they condition the onset of collective phenomena such as synchronization and cascading failures [11,12].

Medicine provides particularly instructive examples because many clinically relevant phenotypes reflect distributed interactions rather than single-locus causation. Systems biology argues that biological function and dysfunction emerge from the dynamical organization of interacting genes, proteins, metabolites, and signaling pathways, and it foregrounds robustness, modularity, and feedback control as central explanatory targets [13]. Network medicine extends this perspective to clinical phenotypes by conceptualizing diseases as perturbations of network modules and by emphasizing that comorbidity, pleiotropy, and shared molecular mechanisms can be represented in network space [14]. This approach shifts emphasis from isolated biomarkers to disease-associated interaction patterns and, correspondingly, from single-target interventions toward strategies that account for network context, pathway crosstalk, and compensatory rewiring.

Complexity is equally evident at the level of organismal physiology, where healthy regulation frequently entails irregular, scale-rich variability rather than steady constancy. Analyses of heart rate and other physiological time series reveal long-range correlations and fractal scaling in healthy subjects, whereas disease and aging can be associated with an erosion of these multiscale patterns [15]. Such findings have encouraged the use of scaling analysis, nonlinear time-series methods, and stochastic modeling to characterize regulatory dynamics and to operationalize “loss of complexity” as a quantitative marker of dysregulation, while also highlighting that the relevant signatures can depend on the observable, the time scale, and the physiological subsystem under study.

Natural systems provide canonical cases in which local interactions generate global order. Ecosystems combine nonlinear population dynamics with networked interactions (predation, competition, mutualism) that can stabilize communities or predispose them to cascading failure; changes in interaction strengths or topology can induce regime shifts that are difficult to anticipate from isolated pairwise mechanisms. At the behavioral level, collective animal motion—bird flocks, fish schools, and insect swarms—illustrates how local rules for alignment, attraction–repulsion, and information transfer can yield coherent group-level patterns such as milling, flocking, and rapid collective turns. These systems exemplify the general complex-systems motif that macroscopic coordination can arise without centralized control, and they motivate models in which local coupling and network structure jointly determine global regimes [16,17].

In physics, complex-systems reasoning is tightly linked to nonequilibrium statistical mechanics, pattern formation, and critical phenomena. Dissipative-structure theory associates macroscopic order with sustained fluxes and entropy production, yielding organized spatiotemporal states (chemical oscillations, convection patterns, reaction–diffusion structures) through instabilities and nonlinear coupling [18,19]. A complementary theme is criticality and scale invariance. The proposal of self-organized criticality suggests that certain driven, dissipative systems naturally evolve toward critical states characterized by power-law event statistics and long-range correlations, providing one route to explaining ubiquitous bursty dynamics and “1/f-like” behavior [20].

Because complex systems span multiple descriptive levels, their mathematical modeling is necessarily pluralistic. A foundational approach uses nonlinear dynamical systems—coupled ordinary or partial differential equations—to represent continuous-state processes with feedback and to analyze attractors, bifurcations, and stability landscapes [18]. When interactions involve discrete events and intrinsic randomness, stochastic process formalisms—master equations, Langevin dynamics, and diffusion approximations—provide a principled way to connect microscopic transition rules to mesoscopic statistics [21]. Many applications are naturally networked, motivating models in which node states evolve on complex graphs and where spreading, percolation, or synchronization depend on both dynamics and topology [11]. Coupled-oscillator models offer a paradigmatic bridge: the Kuramoto model captures how weak coupling yields an order-parameter transition to collective synchrony [22]. When heterogeneity and local rules are central, agent-based models enable simulation of emergent macroscopic regimes from micro-level interaction rules [23].

Taken together, these perspectives motivate an integrative view of complex systems as systems in which macroscopic behavior is shaped by nonlinear interactions among many components, mediated by structured connectivity, organized across multiple scales, and often modified by adaptation or learning. The contemporary challenge is to make this concept operational and predictive: to identify effective coarse-grained variables, infer interaction rules and network structure from data, quantify uncertainty in multiscale models, and design interventions that target system organization rather than isolated component effects [24].

The primary limitation of traditional calculus in analyzing complex systems is its assumption of “smoothness.” If you zoom in on a smooth curve, it eventually looks like a straight line. But complex systems exhibit fractal geometry: no matter how much you zoom in on the data—whether it is a time series of internet traffic or the velocity of turbulent wind—the “roughness” remains [1].

These trajectories are continuous—the system moves from state A to state B without teleporting—but they are nowhere differentiable. In mathematical terms, this resembles the Weierstrass function, a pathological curve that is connected everywhere but has no defined slope anywhere [25,26]. This “jaggedness” is not noise to be filtered out; it is the very signature of the system’s memory and correlations. It implies that the system does not have a single characteristic scale. Instead, fluctuations occur at all scales simultaneously, linking the microscopic interactions to macroscopic behaviors.

While a simple fractal (monofractal) describes a system with a consistent degree of roughness (characterized by a single number, the fractal dimension), complex systems are rarely so uniform. They exhibit intermittency—periods of relative calm punctuated by sudden, extreme bursts of activity.

To describe this heterogeneity, we use multifractal analysis. Imagine the dynamics space not as a uniform shape, but as an interwoven tapestry of many different fractals, each with its own scaling rule. Instead of a single dimension, we calculate a “spectrum” of dimensions (often denoted as *f*(*α*)). This spectrum acts as a fingerprint of the system. The variable *α* (the Hölder exponent) measures the local “regularity” or roughness of the signal at a specific point. A low α indicates violent, sharp bursts (like a market crash), while a high *α* indicates smoother, more correlated behavior [27].

In the present paper, a new approach for analyzing complex systems dynamics is presented, by assimilating the complex system with a multifractal. In such a context, the dynamics of any complex system’s structural units are described through continuous and non-differentiable curves (multifractal curves that simultaneously admit various fractal dimensions).

## 2. Complex Fluids Dynamics in a Multifractal Space—Mathematical Model

Let us accept the functionality of the following scale covariance principle (see [28,29]) in the dynamics of complex systems: the laws of dynamics of any complex system remain invariant, both in relation to space-time coordinate transformations and in relation to scale resolution transformations. In these conditions, the specific momentum conservation law can be assimilated to the geodesics equation in a multifractal space, so that it takes the following form:

(1) $$\frac{\hat{d}{\hat{V}}^{i}}{dt}=\partial_{t}{\hat{V}}^{i}+{\hat{V}}^{l}\partial_{l}{\hat{V}}^{i}+\frac{1}{4}{\left(dt\right)}^{\left[\frac{2}{f(\alpha )}\right]-1}T^{lk}\partial_{l}\partial_{k}{\hat{V}}^{i}=0\;$$

where

$$\partial_{t}=\frac{\partial }{\partial t},\;\;\partial_{l}=\frac{\partial }{\partial x^{l}},\;\;\partial_{l}\partial_{k}=\frac{\partial }{\partial x^{l}}\frac{\partial }{\partial x^{k}},\;\;l,k=1,2,3$$

In Equation (1), ${\hat{V}}^{i}$ is the complex velocity,

$${\hat{V}}^{i}=\frac{\hat{d}X^{i}}{dt}=V_{D}^{i}-{iV}_{F}^{i},\;i=\sqrt{-1},$$

with $V_{D}^{i}$ the differential velocity (dt scale resolution independent), $V_{F}^{i}$ is the non-differential velocity (dt scale resolution dependent), $T^{lk}$ is the tensor coefficient associated with the multifractal-non-multifractal transition, and f(α) is the singularity spectrum (with $\alpha =\alpha \left(D_{F}\right)$ being the singularity index, which is dependent on the motion curves’ fractal dimension $D_{F}$ [30]).

In such context, it results that the multifractal acceleration, $\partial_{t}{\hat{V}}^{i}$, the multifractal convection, ${\hat{V}}^{l}\partial_{l}{\hat{V}}^{i}$ and the multifractal dissipation $T^{lk}\partial_{l}\partial_{k}{\hat{V}}^{i}$ make their balance in any point of the multifractal curve.

Now, separating the complex system dynamics on scale resolutions (differentiable and non–differentiable) (1) becomes

(2) $$\partial_{t}V_{D}^{i}+V_{D}^{l}\partial_{l}V_{D}^{i}-V_{F}^{l}\partial_{l}V_{F}^{i}+\frac{1}{4}{\left(dt\right)}^{\left[\frac{2}{f(\alpha )}\right]-1}T^{lk}\partial_{l}\partial_{k}V_{D}^{i}=0\;\partial_{t}V_{F}^{i}+V_{F}^{l}\partial_{l}V_{D}^{i}+V_{D}^{l}\partial_{l}V_{F}^{i}+\frac{1}{4}{\left(dt\right)}^{\left[\frac{2}{f(\alpha )}\right]-1}T^{lk}\partial_{l}\partial_{k}V_{F}^{i}=0$$

In our proposed multifractal framework, the advantage of using a singularity spectrum f(α) is that it replaces a single “average” roughness descriptor (one fractal dimension) with a full distribution of local scaling behaviors, thereby capturing the heterogeneity and intermittency that drive complex-system dynamics. Concretely, α indexes the local regularity (from sharp bursts to smoother, correlated segments), while f(α) quantifies how much of the trajectory exhibits each α; the resulting spectrum acts as a fingerprint of the system’s multiscale organization and “structural richness.” In the model, this is not just descriptive: the singularity spectrum enters the multifractal geodesic dynamics (via the scale-dependent terms), so it provides an operational way to encode how fluctuations across scales contribute to macroscopic behavior—e.g., distinguishing regimes where the system is effectively monofractal (narrow spectrum) from regimes capable of self-structuring and richer evolution (wide spectrum).

This particular methodology for examining complicated fluid dynamics has been effectively employed to analyze various representations of real fluids, such as blood [31], complex polymers [32], discharge [33], and laser-produced plasmas [34] inside the fluid model framework. The significant outcomes of the non-differentiable approach pertain to the interpretation of the influences exerted by the constituent particles, referred to as structural units of our complex fluid, on the overall dynamics of the complex fluid. We concentrate on how the entire system illustrates the interconnection and interactions of its constituent elements, specifically how the flow of a real fluid is influenced by the interactions among its structural units—atoms, molecules, clusters, nanoparticles, etc. A significant issue in many of their applications is the segregation and organization of the actual fluid (in the depiction of vapor, plasma, polymer, or liquids) during various technological processes. The model seeks to demonstrate that the structuring processes, irrespective of fluid type, are manifestations of same physical forces at varying scale resolutions.

The specifics of the model have been previously addressed in prior publications by our group [31,34]. In summary, we assert that the motion of the complex fluid constituents (structural units) is delineated within certain boundaries by continuous yet non-differentiable curves. This enables us to enhance the projection of real fluid properties within a fractal matrix, so simplifying the dynamics of individual particles or molecules by correlating them with their corresponding fractal geodesics (trajectories). Consequently, over extended time scales relative to the inverse of the maximum Lyapunov exponent [35,36], deterministic trajectories are replaced by collections of possible trajectories (i.e., fractal geodesics), and the notion of defined positions is substituted with that of probability densities.

In such a context, in agreement with the results from [31,37], at a differentiable resolution scale the complex systems dynamics are driven by the specific fractal force:

(3) $$F_{F}^{i}=\left[V_{F}^{l}+\frac{1}{4}{\left(dt\right)}^{\left[\frac{2}{f(\alpha )}\right]-1}T^{kl}\partial_{k}\right]\partial_{l}V_{F}^{i}$$

We want to mention that the fractal force is not an external body force added ad hoc to the momentum equation. It appears when the scale-covariant geodesic dynamics are decomposed into differentiable and non-differentiable scale resolutions (Equation (2)). In this sense, Equation (3) represents the back-reaction of scale-dependent, intermittent fluctuations on the differentiable flow, i.e., an effective force (or, equivalently, an effective stress/pressure-like contribution) induced by the multifractal geometry encoded through the singularity spectrum f(α). In complex-fluid applications this term drives redistribution and segregation of the state density and velocity field across scales, thereby selecting preferential channels and self-structured patterns, as we will show in the present work.

The presence of this specific fractal force in explicit manner could be responsible for the separation of complex fluid on each component, by introducing a special velocity field. To this end we admit the functionality of our differential system of equations:

(4) $$F_{F}^{i}=\left[V_{F}^{l}+\frac{1}{4}{\left(dt\right)}^{\left[\frac{2}{f(\alpha )}\right]-1}T^{kl}\partial_{k}\right]\partial_{l}V_{F}^{i}=0$$

(5) $$\partial_{l}V_{F}^{l}=0$$

The first equation specifies the fact that for a differential scale resolution the multifractal force becomes null, while the second one represents the state density conservation law at non-differentiable scale resolution.

In general it is difficult to obtain an analytic solution for our system of equations, taking into account its non-linear nature (through the multifractal convection $V_{F}^{l}\partial_{l}V_{F}^{i}$ and the multifractal-type dissipation $T^{kl}\partial_{l}\partial_{k}V_{F}^{i}$) and the fact that the fractalization type, given by the multifractal-type tensor $T^{kl}$, is left unknown in these representations.

To advance our model and its application in the analysis of real or abstract fluids, we will delineate the flow of a three-dimensional fluid exhibiting rotational symmetry about the z-axis and examine its dynamics via the two-dimensional projection in the (*x*,*y*) plane.

By considering only the symmetry plane (*x*,*y*), the Equations (4) and (5) system becomes:

(6) $$V_{Fx}\frac{\partial V_{Fx}}{\partial x}+V_{Fy}\frac{\partial V_{Fx}}{\partial y}=\frac{1}{4}{\left(dt\right)}^{\left[\frac{2}{f(\alpha )}\right]-1}\;T^{yy}\frac{\partial^{2}V_{Fx}}{\partial y^{2}}$$

(7) $$\frac{\partial V_{Fx}}{\partial x}+\frac{\partial V_{Fy}}{\partial y}=0$$

where we only used the $T^{yy}$ component of the $T^{kl}$ tensor.

Now, let us solve the equation system (6) and (7) by imposing the following conditions

(8) $$\underset{y\to 0}{lim}V_{Fy}\left(x,y\right)=0,\;\underset{y\to 0}{lim}\frac{\partial V_{Fx}}{\partial y}=0,\;\underset{y\to \infty }{lim}V_{Fx}\left(x,y\right)=0$$

$$\Theta =\int_{-\infty }^{+\infty }{V_{Fx}}^{2}dy=\mathrm{const}.$$

with:

(9) $$T^{yy}=a\exp\left(i\theta \right)$$

where usually, a can have the significance of an amplitude and θ can have the meaning of a phase. For details involving this method, see [28,29].

We would also like to note that the presence of the complex phase can lead to a hidden temporal evolution of the system. It is known that the variation of a complex phase implicitly defines a time dependence which would make our system capable of depicting both space and time evolutions. Thus, the choice for *T^yy^* can lead to the possibility of a both spatial and temporal study on the dynamics of our system.

The solution of Equations (5) and (6), in their outmost general form in the normalized quantities:

(10) $$\;X=\frac{x}{x_{0}},Y=\frac{y}{y_{0}},U=V_{Fx}\frac{4y_{0}^{2}}{x_{o}a},V=V_{Fy}\frac{4y_{0}^{2}}{x_{o}a},\frac{{\left(\frac{\Phi_{0}}{6}\right)}^{\frac{1}{3}}}{{\left(\frac{a}{4}\right)}^{2/3}}=\frac{x_{0}^{2/3}}{y_{0}},\mu =\left[{dt}^{\left(\left[\frac{2}{f(\alpha )}\right]-1\right)}\right]$$

is given according to the method from [38]:

(11) $$U\left(X,Y\right)=\frac{\frac{3}{2}}{{\left[\mu X\right]}^{\frac{1}{3}}\exp\left(\frac{i\theta }{3}\right)}\cdot {\mathrm{sech}}^{2}\left\{\frac{\frac{1}{2}Y}{{\left[\mu X\right]}^{\frac{2}{3}}\exp\left(\frac{2i\theta }{3}\right)}\right\}$$

(12) $$V\left(X,Y\right)=\frac{{\left(\frac{9}{2}\right)}^{\frac{2}{3}}}{{\left[\mu X\right]}^{\frac{1}{3}}\exp\left(\frac{i\theta }{3}\right)}\left\{\left[\frac{Y}{\mu X^{\frac{2}{3}}\exp\left(\frac{2i\theta }{3}\right)}\right]\cdot {sech}^{2}\left[\frac{\frac{1}{2}Y}{{\left[\mu X\right]}^{\frac{2}{3}}\exp\left(\frac{2i\theta }{3}\right)}\right]-\tanh\left[\frac{\frac{1}{2}Y}{{\left[\mu X\right]}^{\frac{2}{3}}\exp\left(\frac{2i\theta }{3}\right)}\right]\right\}$$

**Figure 1:**
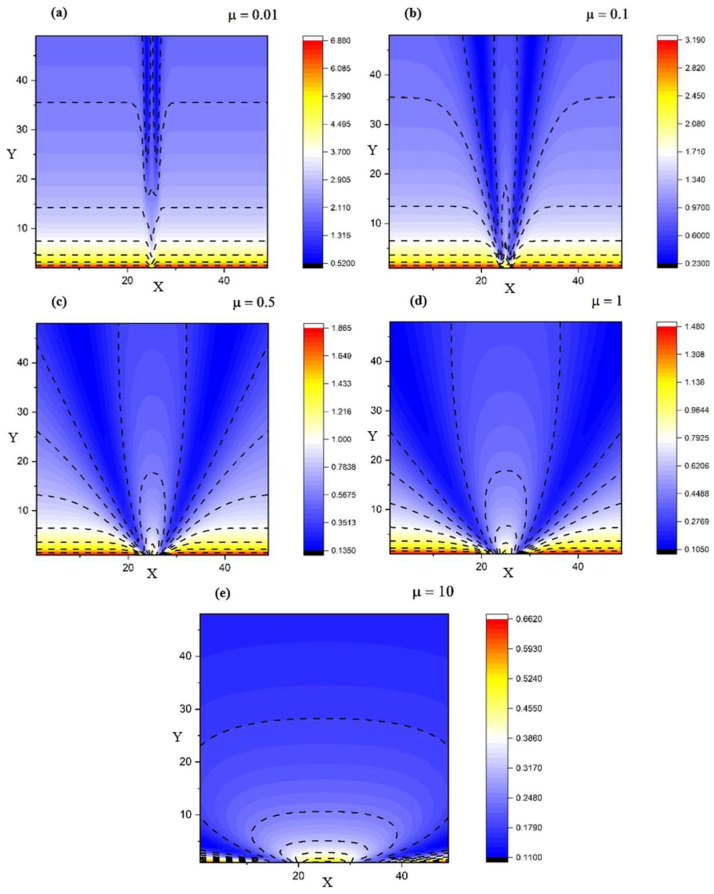
Velocity field variations for different fractality degrees (*µ*—a control parameter): 0.01 (**a**), 0.1 (**b**), 0.5 (**c**), 1 (**d**), and 10 (**e**).

To verify the validity of such unusual approach we obtained 2D modelling representations depicting the flow of a complex fluid based on the solution given by our system of equations. The fluid is defined in the framework of our model as a mixture of various particles with different physical properties. As a result, parameters such as the complex phase, fractal dimension or specific length (*x*_0_, *y*_0_) will incorporate within their values the unique properties of each component. Figure 1 illustrates the effect of a fractality-control parameter *µ* on flow self-structuring: smaller *µ* yields a more collimated central jet, while larger *µ* promotes stronger lateral spreading and smoother streamline bundles.

**Figure 2:**
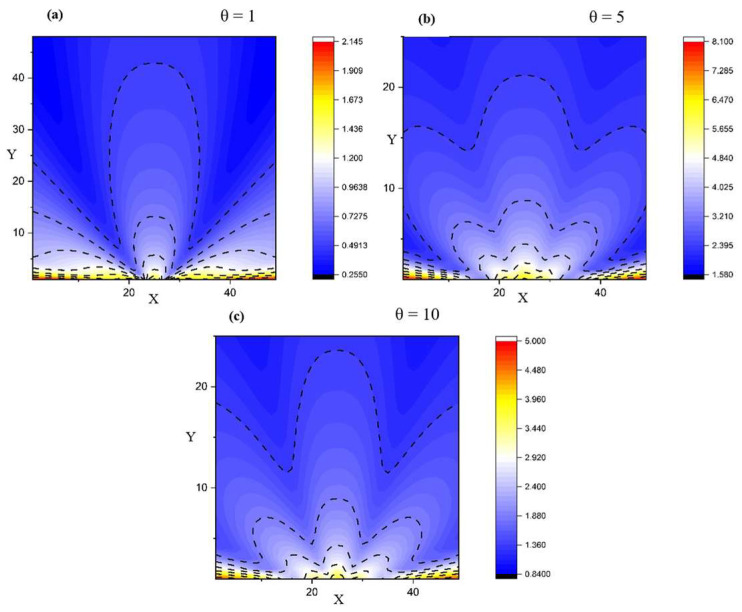
Velocity field variations for different resolution scales for a multifractal system with *θ* having values of 1 (**a**), 5 (**b**), and 10 (**c**).

**Figure 3:**
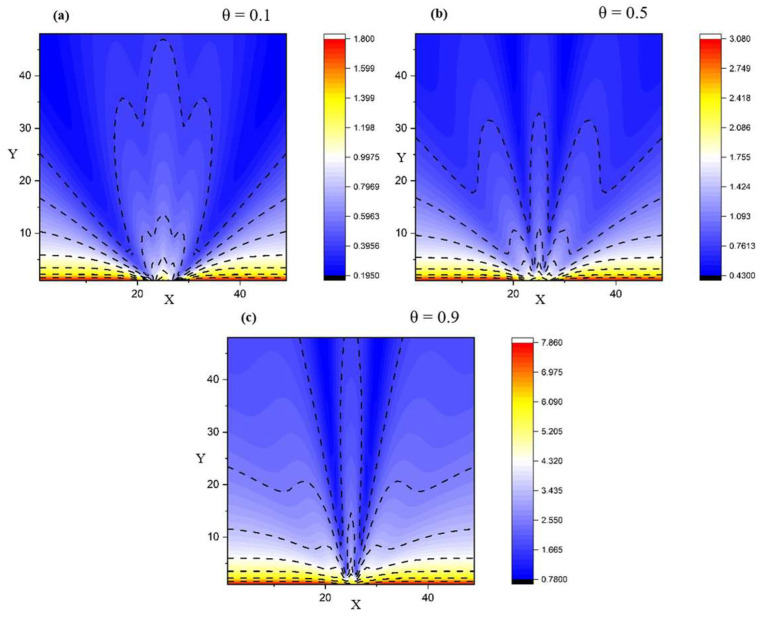
Velocity field variations for different resolution scales for a multifractal system with *θ* having values of 0.1 (**a**), 0.5 (**b**), and 0.9 (**c**).

Figure 2 and Figure 3 show the 2D representations depicting various flow scenarios with respect to the structure of the fluid, starting from a single-particle fluid towards a multi-component fluid. A segregation into multiple structures in all expansion directions (across *X* and *Y*) can be seen. We observe that the self-structuring process is gradual. For different values of *θ*, we obtain various structures.

**Figure 4:**
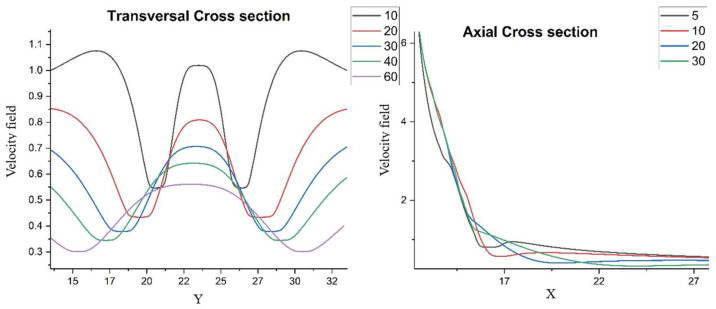
Cross section at various spatial scales for the velocity field generated for a fractality degree of 1.

Figure 4 complements the 2D maps by making the self-structuring regimes explicit in 1D. In the transversal cuts, the near-field profile is strongly non-uniform (multiple extrema), consistent with the preferential channeling seen in the streamline plots; with increasing *X* the profiles relax and the contrast between maxima and minima decreases. In the axial cuts, the velocity field decreases rapidly away from the source for all *Y*, indicating that the dominant transport/structuring mechanisms are set in the near-field through the balance of multifractal convection and multifractal-type dissipation terms (Equation (2)).

## 3. Discussions

The transition from a reductionist, “clockwork” worldview to one of complexity necessitates a fundamental re-evaluation of how we measure the state of a system. In classical mechanics, a system’s future is determined by its initial conditions and the smooth, differentiable laws that govern it. In our proposed model, complex systems operate on continuous but non-differentiable curves. This “roughness” is not merely an aesthetic feature; it is a reservoir of information [39,40]. Here, the concept of entropy becomes the bridge between the deterministic microscopic world and the unpredictable macroscopic reality.

In the context of our multifractal model, entropy is not simply a measure of disorder, as defined in classical thermodynamics, but rather a metric of the system’s structural richness and its potential for self-organization. When we describe a system’s dynamics through a singularity spectrum f(α), we are essentially mapping its entropic potential. The variable α measures the local regularity—how “smooth” or “rough” the behavior is at a specific point—while f(α) describes the dimension of the subset of points having that specific α. In information theory terms, this spectrum quantifies the density of information stored in the system’s fluctuations [41,42]. A wide f(α) spectrum indicates a system rich in complexity, capable of accessing a vast number of states, whereas a narrow spectrum suggests a system locked into a repetitive, monofractal behavior.

This perspective can shift our understanding of “equilibrium.” In standard physics, systems tend toward maximum entropy. However, complex adaptive systems—like a living cell or a global market—maintain themselves in a state of “dynamic instability”. They exist at the “edge of chaos,” a sweet spot between the stagnation of total order and the incoherence of total randomness [43]. Our mathematical derivation of the “fractal force” (Equation (3)) supports this view. The fractal force is not an external push; it acts as an intrinsic “entropic force” arising from the scale-dependence of the system. It drives the “self-structuring” processes we observed in our simulations, where the fluid spontaneously separates into preferential flow lines or structures without a “master” controller. The “state density conservation law” at the non-differentiable scale implies that while the system evolves, the information encoded in its multifractal geometry is preserved and transformed, guiding the emergence of global patterns from local interactions [44,45,46].

The power of this non-differentiable approach lies in its universality. By assimilating the dynamics of any complex system unit to a multifractal curve, we create a “common language” that transcends disciplinary boundaries. This allows us to apply our model to fields as diverse as medicine, atmospheric physics, and geophysics, where standard differential calculus often fails to capture reality.

In the medical field, the distinction between “complicated” and “complex” is often a matter of life and death. Human physiology is the ultimate complex system, where “health” is characterized by a high degree of multifractal complexity—think of the irregular yet rhythmic beating of a healthy heart or the branching capability of a neural network. Disease often manifests as a loss of this complexity or a shift toward pathological order [15,47].

Consider cancer dynamics. A tumor is not merely a collection of rapidly dividing cells; it is a complex system that engineers its own environment. The vascular network of tumors is chaotic and characterized by a different fractal dimension compared to healthy tissue. While healthy vessels follow an efficient, hierarchical branching pattern (robustness), tumor vessels exhibit “percolation-like” growth—a disorganized mesh that inhibits efficient transport [48,49]. Our model’s ability to describe “intermittency” and “extreme bursts of activity” is particularly relevant here. The tumor’s growth is not smooth; it occurs in intermittent spurts driven by the non-linear feedback loops of angiogenesis factors. By analyzing the singularity spectrum of tumor vascularization, clinicians could potentially distinguish between aggressive and dormant tumors based on their “roughness” signature, long before macroscopic changes become visible.

Furthermore, our focus on complex fluids has direct implications for controlled drug release. Modern medicine increasingly relies on delivering drugs through complex polymer matrices. The release of a drug from such a matrix is not a simple linear diffusion process (Fickian diffusion [50]); it is a “fractal walk” through a labyrinth of pores and channels [51]. The drug molecules interact with the polymer chains, which we can model as “structural units” with their own fractal geodesics. As our model suggests, the “fractal force” effectively separates the flow components, meaning the drug release rate is governed by the multifractal geometry of the matrix. Understanding this allows for the design of “smart” delivery systems [52,53] where the release profile is tuned by altering the fractal dimension of the polymer carrier, ensuring a steady therapeutic dose rather than a toxic burst [54,55].

Moving from the microscopic to the macroscopic, the atmosphere presents one of the most challenging complex systems known to science. Traditional meteorology relies on the Navier–Stokes equations, which assume smooth, continuous fluid flow [56]. However, anyone who has experienced a sudden drop in altitude on a flight knows that the atmosphere is far from smooth. It is dominated by turbulence—a phenomenon that is inherently multifractal.

In our model, the “velocity of turbulent wind” can be treated as a non-differentiable curve. The “jaggedness” of the wind speed data is not noise; it is the signature of energy cascading from large weather systems down to microscopic eddies [57]. The “singularity spectrum” could be used to characterize this turbulence. A specific value of f(α) can identify “laminar channels” or stable zones within a chaotic storm, while a different value points to areas of extreme shear and unpredictability. This aligns with the concept that “new laws and behaviors appear” at different scales. By applying the multifractal potential equations (Equations (1) and (2)), atmospheric physicists can better model these phase transitions, improving predictions for extreme weather events that standard linear models often miss.

Finally, the non-differentiable perspective offers profound insights into geophysics, particularly in the study of earthquakes. Earth’s crust is a network of fault lines that are self-organized into a critical state. An earthquake is not an isolated random event; it is a system-wide failure resulting from long-term correlations and “memory” stored in the rock’s stress field [58]. Standard calculus cannot easily model the jagged, discontinuous rupture of a fault. However, by viewing the fault system as a multifractal timeline, we can analyze the “intermittency” of seismic silence punctuated by violent release. The “fractal force” in our model helps explain how a tiny perturbation—a “butterfly effect”—can be amplified through positive feedback loops to trigger a massive quake. Research suggests that the singularity spectrum of seismic noise changes prior to a major event [59]. By monitoring the “breathing modes” of the crust—the oscillatory shifts in its fractal dimension—geophysicists may one day identify the “hidden temporal evolution” that precedes a catastrophic shift, moving from simple probability to genuine forecasting.

### 3.1. Relation to Non-Extensive Statistical Mechanics (Tsallis Statistics)

Our multifractal–geodesic formulation is naturally aligned with the non-extensive statistical mechanics formalism introduced by Tsallis, where the entropic functional $S_{q}$ generalizes Boltzmann–Gibbs entropy and was originally motivated (in part) by multifractal geometries and long-range correlations [60]. In this view, the singularity spectrum f(α) implies a hierarchy of generalized (Rényi) dimensions $D_{p}$ and corresponding generalized entropies; Tsallis’ q-index provides an alternative parametrization of departures from the q=1 (extensive) limit [61,62,63,64]. Moreover, for systems at the edge of chaos, a direct link exists between the entropic index associated with sensitivity to initial conditions and the extrema of the multifractal spectrum: $1/\left(1-q_{sen}\right)=1/\alpha_{min}-1/\alpha_{max}$ [62]. While the present paper does not attempt a full *q*-triplet estimation, this relation suggests a practical route for connecting our inferred f(α) to *q*-statistics: (i) extract f(α) from the data and estimate $q_{sen}$; (ii) estimate $q_{stat}$ by fitting stationary probability density functions of increments with *q*-Gaussians; and (iii) estimate $q_{rel}$ from relaxation laws of correlation functions or return-to-equilibrium observables [61].

### 3.2. Practical Parameter Identification from Data

In the following, we outline an operational workflow for inferring the central model inputs (*μ*, *θ* and the singularity spectrum f(α)) from experimental observations. The key idea is to treat parameter identification as an inverse problem in which multifractal diagnostics are extracted from data and then used to constrain (or initialize) a fit of the analytical/numerical solutions of Equations (6) and (7) [65,66,67]:

- Data pre-processing: detrend, remove obvious non-stationarities, and select the scale range over which scaling is expected (e.g., inertial range for turbulence).
- Estimate f(α): for 1D time series, use Multifractal Detrended Fluctuation Analysis [65] or Wavelet Transform Modulus Maxima [68] to obtain scaling exponents and perform the Legendre transform to recover f(α). For images (e.g., vascular networks), use multifractal box-counting/partition function methods on the intensity or skeletonized measure.
- Extract summary descriptors from f(α): endpoints ($\alpha_{min}$, $\alpha_{max}$), peak position $\alpha_{0}$, and spectrum width $∆\alpha =\alpha_{max}-\alpha_{min}$; these quantify intermittency/heterogeneity and can be reported alongside $D_{F}$ (or related generalized dimensions).
- Calibrate *μ* and *θ* against macroscopic observables of the modeled field: in our 2D jet-like solutions, *μ* primarily controls collimation vs. lateral spreading, while *θ* controls the degree of multi-structure segregation with resolution scale; therefore use measurable quantities such as centerline decay, half-width/spreading rate, number of lobes/extrema in transverse cuts, or an integral “structuring index” (e.g., std(*U*)/mean(*U*) across Y).
- Inverse fitting: define an objective function that combines (a) mismatch of the measured f(α) descriptors and (b) mismatch of macroscopic flow statistics, then estimate (*μ*, *θ*, …) by nonlinear least squares or Bayesian inference [69]; report confidence intervals and sensitivity.

### 3.3. Minimal Quantitative Benchmark Against Canonical Jet Similarity Laws

**Figure 5:**
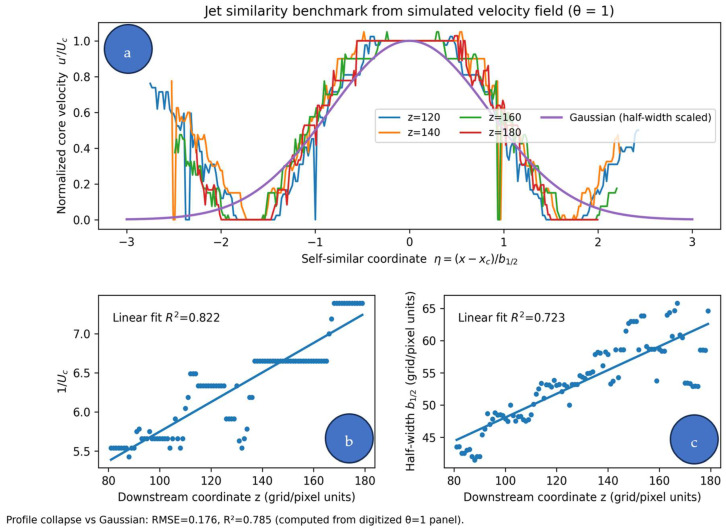
Quantitative benchmark against canonical turbulent round-jet similarity behavior extracted from the simulated *θ* = 1 velocity field. (**a**) Self-similar collapse of normalized transverse profiles using half-width scaling $u\prime /U_{c}\approx exp\left(-ln2\eta^{2}\right)$ with $\eta =\left(x-x_{c}\right)/b_{1/2}$. (**b**) Linear dependence of $1/U_{c}$ on downstream coordinate in the self-preserving region. (**c**) Linear growth of the half-width $b_{1/2}$ with downstream coordinate. Goodness-of-fit metrics are reported in-panel.

A minimal (data-free) benchmark can be performed by comparing the model’s predicted mean-field profiles against established similarity results for turbulent round jets. In the self-preserving region, the normalized axial-velocity profile is well approximated by a Gaussian and the centerline velocity decays approximately as $U_{c}\left(x\right)\propto {\left(x-x_{0}\right)}^{-1}$ while the jet half-width grows linearly with ∝ [70,71,72]. Using the analytical solution (Equations (11) and (12)), one can extract $U_{c}\left(x\right)$ and $r_{1/2}\left(x\right)$, fit the above scalings, and report fit coefficients and goodness-of-fit ($R^{2}$ or RMSE). In addition, overlay the normalized transverse profiles from our model with the Gaussian similarity form and report the profile-wise misfit. This provides a quantitative positioning relative to classical jet behavior and requires only the fields already generated for Figure 1, Figure 2, Figure 3 and Figure 4. Figure 5 provides an example of this benchmark applied to the *θ* = 1 field, showing self-similar profile collapse and the expected linear trends for $1/U_{c}$ and the half-width $b_{1/2}$.

### 3.4. Implications for Processed Hydrodynamic Simulations: Using f(α) and Interpreting the Fractal Force

A common use case is: (i) run a hydrodynamic simulation [73] (Direct Numerical Simulation/Large Eddy Simulation) or obtain experimental fields (e.g., Particle Image Velocimetry [74]), (ii) post-process the velocity (or a derived measure such as dissipation) to estimate a multifractal spectrum *f*(*α*), and then (iii) ask what, physically, the spectrum and the associated “fractal force” add beyond standard turbulence diagnostics. In this setting, the present framework suggests that f(α) is not only a fingerprint of intermittency: within Equations (1)–(3) it parameterizes the scale-dependent part of the dynamics, so changes in the spectrum correspond to changes in the strength and localization of the non-differentiable contribution and of the associated scale-induced forcing term.

Practically, the deeper physical insight comes from connecting f(α) to where the flow is “rough” (low *α*, rare bursts) versus “smooth” (high *α*), and then relating those sets to the structures and budgets that control transport, mixing, and dissipation.

In this interpretation, the fractal force is “forcing” the cross-scale exchange of momentum (and, by extension, energy and scalar variance) between the smooth, resolution-independent component and the intermittent, scale-dependent component. Therefore, once f(α) is available from a simulation, Equation (3) can be evaluated (up to the model’s multifractal coefficients) as a diagnostic field to localize where multiscale activity feeds back on the mean flow, and it provides a concrete target for model validation by comparison with the residual term that appears when Navier–Stokes is filtered at the same scale.

### 3.5. Limitations

We would also like to mention several limitations of our model:

(i) The practical application will depend on robust methods to infer model parameters (e.g., those entering the scale-dependent terms and the singularity spectrum) from experimental or observational data.
(ii) The simulations demonstrate qualitative self-structuring regimes; quantitative validation against measured flows or time series (and benchmarking versus established models) is needed to delimit the model’s predictive power.
(iii) Our work emphasizes universality; however, domain-specific constitutive assumptions (e.g., mapping physical microstructure into θ, μ, and related quantities) will determine whether universality translates into actionable predictions in specific systems.

## 4. Conclusions

In this manuscript, we proposed a multifractal formulation for complex-system dynamics by treating the trajectories of structural units as continuous but non-differentiable curves, thereby making scale dependence a constitutive feature of the model rather than an empirical afterthought. Within the scale-covariance principle, the momentum conservation law is recast as a geodesic equation in a multifractal space, yielding a complex velocity field whose differentiable and non-differentiable components separate naturally and whose balance explicitly involves multifractal acceleration, convection, and dissipation (Equations (1) and (2)). This establishes an operational role for the singularity spectrum f(α): beyond characterizing intermittency and heterogeneity, it directly parametrizes the scale-dependent terms governing macroscopic evolution.

A key theoretical consequence is the emergence of an intrinsic “fractal force” (Equation (3)), interpretable as an entropic/scale-induced driver of self-organization. In the reduced, symmetry-informed 2D setting, the coupled conservation system (Equations (6) and (7)) admits analytic solutions under physically interpretable constraints (Equations (8)–(12)), enabling direct exploration of how fractalization controls flow morphology. The numerical visualizations support the model’s central claim that self-structuring and component segregation can arise without external “master” control: varying the fractality-control parameter μ modulates the transition from collimated jet-like regimes to laterally spreading, smoother bundles (Figure 1), while changes in the resolution-scale parameter θ generate gradual differentiation into multiple preferential structures (Figure 2 and Figure 3). Cross-sectional profiles further indicate that structuring is predominantly set in the near-field—where strong non-uniformity and multiple extrema occur—and then relaxes downstream, consistent with a local balance between multifractal convection and multifractal-type dissipation (Figure 4).

For hydrodynamic applications, this means that once a multifractal spectrum f(α) is extracted from velocity or dissipation fields, the model predicts a corresponding scale-induced forcing term (Equation (3)) that can be mapped in space and time and compared directly with filtered-equation residuals or subgrid stresses.

Conceptually, the work reframes entropy in complex systems as a measure of structural richness encoded in multiscale fluctuations: wide singularity spectra correspond to richer accessible dynamics and stronger self-organization potential, whereas narrow spectra approach effectively monofractal, repetitive regimes. This perspective helps rationalize why complex adaptive systems can persist in dynamically maintained, “edge-of-chaos”-like regimes and clarifies how non-differentiability can store and transform information rather than merely representing measurement noise.

## Data Availability

All the data is presented in the manuscript.
